# Correlation between lumbar multifidus fat infiltration and lumbar postoperative infection: a retrospective case–control study

**DOI:** 10.1186/s12893-019-0655-9

**Published:** 2020-02-24

**Authors:** Chaohui Sang, Xushi Chen, Hailong Ren, Zhandong Meng, Jianming Jiang, Yi Qin

**Affiliations:** 1 Department of Orthopedics, Zhuhai People’s Hospital, NO. 79 Kangning Road, Zhuhai, 519000 Guangdong China; 2 Department of Spinal surgery, Huizhou Municipal Central Hospital, Huizhou, China; 3 Department of Spinal Surgery, Nangfang Hospital, Southern Medical University, Guangzhou, Guangdong China

**Keywords:** Spine, Multifidus fat infiltration, Lumbar postoperative infection, Risk factor, Surgical site infection

## Abstract

**Background:**

The aim of this study was to investigate the correlation between lumbar multifidus fat infiltration and lumbar postoperative surgical site infection (SSI). Several clinical studies have found that spine postoperative SSI is associated with age, diabetes, obesity, and multilevel surgery. However, few studies have focused on the correlation between lumbar multifidus fat infiltration and SSI.

**Method:**

A retrospective review was performed on patients who underwent posterior lumbar interbody fusion (PLIF) between 2011 and 2016 at our hospital. The patients were divided into SSI and non-SSI groups. Data of risk factors [age, diabetes, obesity, body mass index (BMI), number of levels, and surgery duration] and indicators of body mass distribution (subcutaneous fat thickness and multifidus fat infiltration) were collected. The degree of multifidus fat infiltration was analyzed on magnetic resonance images using Image J.

**Results:**

Univariate analysis indicated that lumbar spine postoperative SSI was associated with urinary tract infection, subcutaneous fat thickness, lumbar multifidus muscle (LMM) fat infiltration, multilevel surgery (≥2 levels), surgery duration, drainage duration, and number of drainage tubes. In addition, multiple logistic regression analysis revealed that spine SSI development was associated with sex (male), age (> 60 years), subcutaneous fat thickness, LMM fat infiltration, and drainage duration. Receiver operating characteristic curve analysis indicated that the risk of SSI development was higher when the percentage of LMM fat infiltration exceeded 29.29%. Furthermore, Pearson’s correlation analysis demonstrated that LMM fat infiltration was correlated with age but not with BMI.

**Conclusion:**

Indicators of body mass distribution may better predict SSI risk than BMI following PLIF. Lumbar Multifidus fat infiltration is a novel spine-specific risk factor for SSI development.

## Introduction

Lumbar intervertebral disc herniation accompanied by lumbar instability is the most common cause of low back pain [1, 2]. Lumbar instability can lead to nucleus pulposus protrusion, which can press against nerve roots; this in turn can cause nerve root denaturation and even disability [3]. Therefore, active treatment of lumbar intervertebral disc herniation accompanied by lumbar instability is essential for the prevention of radicular degeneration. Currently, the treatment of this disease primarily involves posterior lumbar interbody fusion (PLIF) [4]. In this PLIF, the diseased intervertebral disc is removed, and the intervertebral fusion device is then implanted in the intervertebral space, which can effectively restore the height of the intervertebral space and the physiological kyphosis of the lumbar spine [2]. However, deep wounds following PLIF are susceptible to infection. Therefore, it is essential to better understand the factors related to lumbar postoperative infection.

Surgical site infection (SSI) is a common complication of spinal surgery, and it is associated with potentially devastating consequences such as neurological injury, sepsis, and death [5, 6]. The morbidity of infection has been reported in 8.5% of primary surgeries and 12% of revision surgeries [7]. Previous retrospective studies have found several risk factors for spine postoperative SSI development, such as diabetes, old age, obesity, poor nutritional status, multilevel surgery, longer surgery duration, and high blood loss [8–15]. The exploration of the risk factors for lumbar spine postoperative SSI following PLIF will therefore provide a basis for formulating SSI prevention measures.

Lumbar multifidus muscle (LMM) is the largest and most medial of the lumbar paraspinal muscles. With LMM degeneration, several age-related changes occur, such as decreased size and increased fat infiltration [16]. LMM fat infiltration occurs when a certain amount of fat tissue replaces the normal architecture of the muscle and accumulates between the LMM and vertebral plate [17, 18]. Numerous studies have reported that fatty infiltration is closely associated with infection [19, 20]. However, few studies have explored the association between LMM fat infiltration and lumbar postoperative SSI. Fat infiltration can occur in LMM; however, the association between fat infiltration and LMM postoperative infection has not yet been reported.

We performed a retrospective case control study for analyzing the risk factors for SSI and determining the correlation between LMM fat infiltration and SSI development in patients undergoing PLIF.

## Materials

### Clinical samples

This study was approved by the Use Committee of the Southern Medical University Nangfang Hospital and Zhuhai People’s Hospital. This retrospective case–control study included patients who underwent PLIF primarily via the midline posterior approach from 2011 to 2016 at our hospital. A total of 3080 patients underwent lumbar surgeries at our hospital from 2011 to 2016; of these, 63 (2.04%) developed postoperative SSI (infection rate, 2.04%). Of the 1426 patients who met the inclusion criteria, 52 patients (32 male and 20 female) who were identified as having SSI within 30 days of surgery comprised the SSI group, and the 1374 patients (667male and 707 female) who did not develop postoperative SSI comprised the non-SSI group; these groups are shown in the flow chart provided as Additional file 1: Fig. S1. All patients included in this study were required to provide complete hospital records and undergo lumbosacral magnetic resonance imaging (MRI) within 2 months before the surgery. Patients with a gross deformity of the spine such as scoliosis or kyphosis, revision surgery, spinal fracture, tumors, tuberculosis of the spine, or spinal infection were excluded from the study.

### Clinical diagnosis

Preoperative and intraoperative data for each patient were collected from their medical records. These data included age, sex, weight and height, presence of diabetes, preoperative albumin (Alb) and hemoglobin (HGB) concentrations, rate of preoperative urinary tract infections (UTIs), number of surgery levels, duration of surgery, blood loss, and the number and duration of closed suction drains.

### SSI assessment

SSI was identified according to the U.S. Centers of Disease Control and Prevention definition [21] and confirmed using a combination of hospital records; laboratory markers; MRI findings; and infection symptoms (such as increased wound drainage, fever, increased pain, or wound erythema) with positive microbiological cultures of specimens from the wound; and/or diagnosis by the surgeon or attending physician. In this study, SSI was defined as an acute infection developing within 30 days of surgery.

### Imaging analysis

**Fig. 1:**
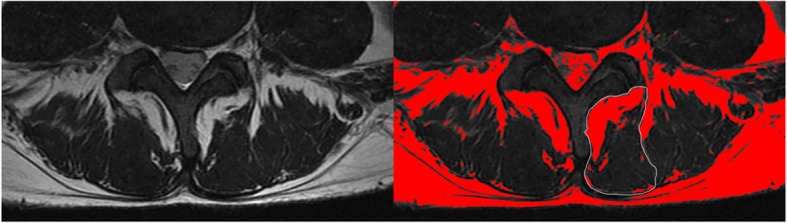
Cross section of the lumbar spine. T2WI MRI and calculation of the cross-sectional area of the multifidus muscles and fat infiltration

MRI (3.0 T, Philips, Best, the Netherlands) of the lumbosacral spine was performed within 2 months before the surgery. Three cross-sectional images of each disc level were available, and the middle image was used to obtain the cross-sectional area (CSA) of LMM in the operative levels using Image J (version 1.40; US National Institutes of Health, Bethesda, MD, USA) [22]. CSA of LMM was defined by outlining the innermost fascial border surrounding the muscle and was inclusive of all fat within the fascial boundary. Fat situated between LMM and the laminae or spinous process was included in CSA of LMM, whereas fat between LMM and the erector spinae muscle was excluded [22]. The area of fat tissue was measured by the thresholding function of the software to obtain image-specific signal intensity ranges for fat tissue in the first outline of LMM (Fig. 1). Measurements were performed separately on the right and left sides. The percentage of LMM fat infiltration was calculated according to the following formula:

$$ \mathrm{LMM}\ \mathrm{fat}\ \mathrm{infiltration}\ \left(\%\right)=\frac{\ \mathrm{CSA}\ \mathrm{of}\ \mathrm{fat}\ \mathrm{tissue}}{\mathrm{CSA}\ \mathrm{of}\ \mathrm{LMM}\ \mathrm{on}\ \mathrm{the}\ \mathrm{same}\ \mathrm{level}\ \mathrm{and}\ \mathrm{side}} \times 100. $$

**Fig. 2:**
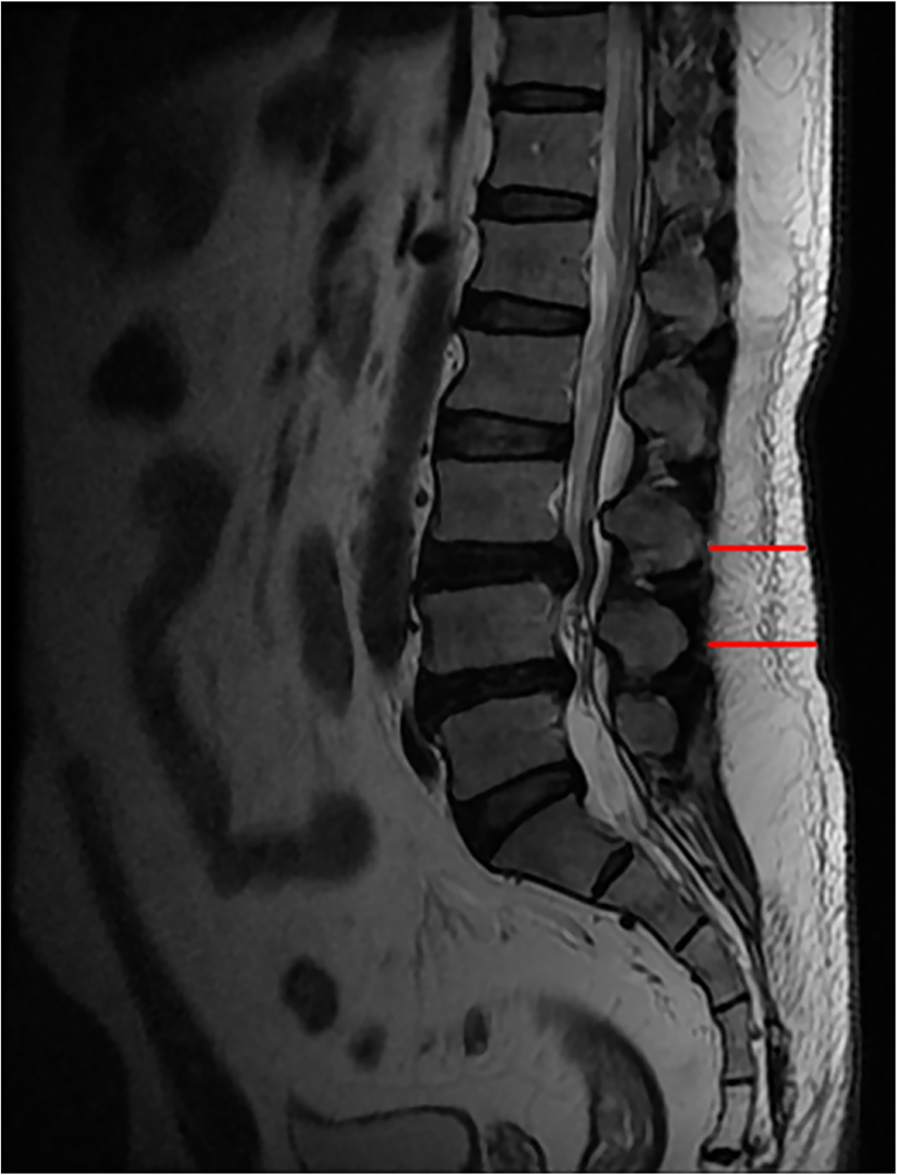
Posterior midline subcutaneous fat measurements at T12–L5 in the sagittal plane of the lumbar spine (T2WI)

The percentage of LMM fat infiltration, which was entered into analysis, was averaged across the left and right sides at the same operative levels. Subcutaneous fat thickness was calculated as the distance from the spinous processes to the back skin in a standardized manner with T2-weighted images reformatted into axial and sagittal orientations (Fig. 2) [23]. All measurements were performed at the operative levels.

### Measurement evaluation

Each measurement was manually verified for accuracy by two orthopedic researchers. Inter-observer Pearson coefficients of *r* = 0.998 (LMM fat infiltration), *r* = 0.991 (subcutaneous fat thickness). No significant difference was observed in the measurements between the evaluators.

### Statistical analysis

Data were analyzed using IBM SPSS Statistics 23 (IBM Corporation, Armonk, NY, USA). Levene’s test was used to determine the homogeneity of the variance of continuous variables. Independent samples t-test was used to compare two groups of continuous variables with equal variances, while the Welch test was used to compare two groups of continuous variables with heteroscedasticity. The χ2 test was used for categorical variables. Pearson’s correlation coefficients were calculated to assess the correlation between two categorical variables. Multivariate logistic regression analysis was performed to determine the independent risk factors for SSI using variables with *p* < 0.1 in the univariate logistic regression analysis. Receiver operating characteristic (ROC) curve analysis was performed to determine the cutoff value. Differences were considered significant at less than 5% (*p* < 0.05).

## Results

### Comparison of demographic data between the groups

Results of comparison of demographic data between the two groups are shown in Table 1 and Additional file 2: Table S1. The mean age of patients was 51.15 years (±14.36) in the SSI group and 51.96 (±12.18) years in the non-SSI group (*p* = 0.692); the two groups did not show any difference in terms of sex (61.54% vs. 48.54%, *p* = 0.066). The other baseline characteristics, such as BMI (*p* = 0.593), presence of diabetes (*p* = 0.292), hypertension (*p* = 0.711), HGB (*p* = 0.118), and preoperative albumin (Alb) level (*p* = 0.759) were also not significantly different. In contrast, the percentage of preoperative UTIs was higher in the SSI group (25%) than in the non-SSI group (13.03%), and this difference was statistically significant (*p* = 0.013). Moreover, patients in the SSI group showed greater subcutaneous fat thickness (15.72 ± 7.27 mm vs. 13.80 ± 7.73 mm, *p* = 0.020) and higher LMM fat infiltration (29.14 ± 18.19% vs. 21.40 ± 10.68%, *p* = 0.005) at the surgical sites. The frequency of multilevel surgery (≥2 levels) was higher in the SSI group (50%) than in the non-SSI group (34.86%), and this difference was statistically significant (*p* = 0.025). Patients with SSI required a longer surgery duration than uninfected patients (209.38 ± 54.27 min vs. 184.15 ± 53.52 min, respectively), and this difference was also statistically significant (*p* < 0.001). The other indexes, such as blood loss (265.38 ± 154.63 ml vs. 260.44 ± 226.42 ml, *p* = 0.876) and drainage volume (328.06 ± 197.57 ml vs. 295.96 ± 172.81 ml, *p* = 0.079), were not significantly different between the two groups. However, there was a higher proportion of patients with 1 drainage tubes (*p* = 0.027) and longer drainage duration than those in the non-SSI group (*p* = 0.037) (Table 1). Therefore, the following statistically significant risk factors may be associated with SSI development: preoperative UTI, higher percentage of fat infiltration, multilevel surgery (≥2 levels), longer surgery duration, less drainage tubes, and longer drainage duration.

**Table 1:** Comparison of Demographic Data between Infection and No-infection Patients

Demographic data	Group		*P*	Levene test	
	SSI Group (*n* = 52)	Non-SSI Group (*n* = 1374)		*F*	*P*
Age	51.15±14.36	51.96±12.18	0.692	3.43	0.064
Male gender	32 (61.54%)	667 (48.54%)	0.066		
BMI	24.54±3.59	24.27±3.44	0.593	0.07	0.796
UTI	13 (25%)	179 (13.03%)	0.013*		
Diabetes	6 (11.54%)	104 (7.57%)	0.292		
Hypertension	10 (19.23%)	237 (17.25%)	0.711		
HGB	140.79±13.72	137.26±16.07	0.118	1.50	0.220
Alb	40.82±2.66	40.70±3.44	0.759	3.97	0.046
Subcutaneous fat thickness	15.72±7.27	13.80±7.73	0.020*	0.23	0.632
LMM Fat infiltration	29.14±18.19	21.40±10.68	0.005*	19.51	<.0001
Multilevel surgery(≥2 levels)	26 (50%)	479 (34.86%)	0.025*		
Surgery duration	209.38±54.27	184.15±53.52	<.001*	0.00	0.980
Bleeding	265.38±154.63	260.44±226.42	0.876	0.10	0.748
The volume of drainage	328.06±197.57	295.96±172.81	0.079	0.51	0.473
The duration of drainage	2.38±0.82	2.14±0.45	0.037*		
The number of drainage tubes	1 (79.84%)	1 (67.31%)	0.027*		
	2 (20.16%)	2 (32.69%)			

*UTI* urinary tract infection, *BMI* body mass index, *LMM* lumbar multifidus muscle, *HGB* hemoglobin, *Alb* albumin

*Statistically significant

### Multivariate logistic regression analysis of risk factors for SSI development

Univariate logistic regression analysis of risk factors showed that patients in the SSI group showed higher LMM fat infiltration (*p* < 0.001) and longer drainage duration than those in the non-SSI group (*p* = 0.001). According to multivariate logistic regression analysis, male sex (OR = 4.55, 95% CI = 2.05–10.06 for male, *p* < 0.001) and age were significant risk factors (OR = 2.36, 95% CI = 1.06–5.29 for age > 60 years, *p* = 0.038). In addition, higher percentage of LMM fat infiltration (OR = 1.06, 95% CI = 1.03–1.08, *p* = 0.000), greater subcutaneous fat thickness (OR = 1.06, 95% CI = 1.02–1.10, *p* = 0.004), and longer drainage duration (OR = 2.24, 95% CI = 1.15–4.36, *p* = 0.018) were independent risk factors for SSI development (Table 2).

**Table 2:** Univariate and Multiple Logistic Regression Analysis of Risk Factors for the Development of Spine Surgical Site Infections

	Univariate Logistic Regression		Multiple Logistic Regression	
Risk Factors	Odds ratio (95% Wald CI)	*P*	Odds ratio (95% Wald CI)	*P*
Sex (male)	1.696 (0.0.96,3.00)	0.069	4.55 (2.05,10.06)	0.000*
Age(>60 yrs)	1.103 (0.59,2.06)	0.758	2.36 (1.06,5.29)	0.038*
Subcutaneous fat thickness	1.03 (0.99,1.07)	0.092	1.06 (1.02,1.10)	0.004*
LMM Fat infiltration ^a^	1.53 (1.27,1.84)	0.000*	1.73 (1.1.34, 1.2.23)	0.000*
The duration of drainage	1.81 (1.28,2.55)	0.001*	2.24 (1.15,4.36)	0.018*

*Statistically significant; ^a^ we set every 10% change in fat infiltration into analysis. LMM: lumbar multifidus muscle

### Linear regression analysis of the association of LMM fat infiltration with BMI and age

Linear regression analysis of the association between LMM fat infiltration with BMI and age revealed that LMM fat infiltration was correlated with age (r = 0.128, *p* = 0.001) but not with BMI (r = 0.002, *p* = 0.183) according to Pearson’s correlation analysis (Table 3).

**Table 3:** Linear Regression Analysis of LMM Fat Infiltration with BMI and Age

	Sample size	Correlation coefficient	*P*
BMI	1426	0.002	0.183
Age	1426	0.128	<0.001^a^

^a^Statistically significant

### ROC curve analysis of the association between LMM fat infiltration and SSI

**Fig. 3:**
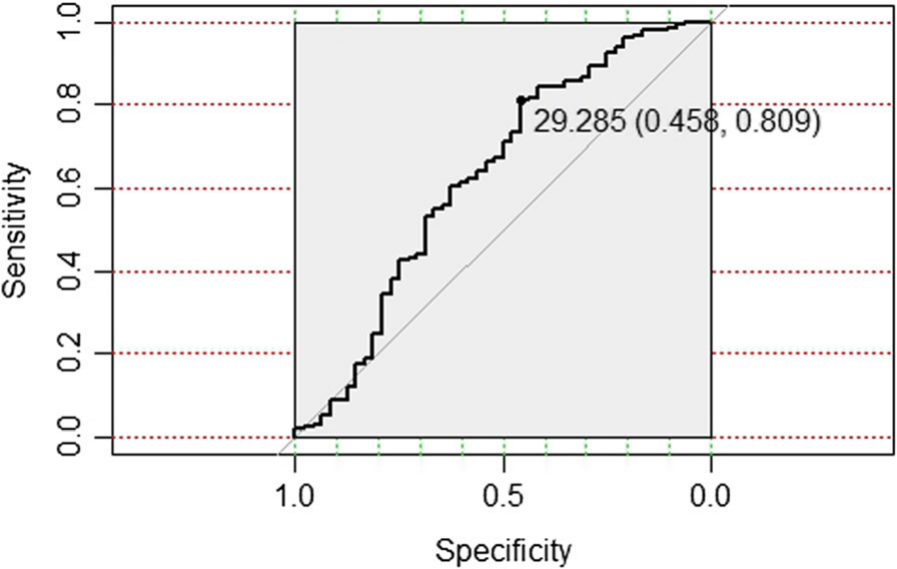
Receiver operating characteristic (ROC) curve analysis of LMM fat infiltration in SSI

ROC curve analysis was performed to predict the association between SSI and LMM fat infiltration. The ROC curve indicated that the risk of SSI was higher when the percentage of LMM fat infiltration exceeded 29.29% (sensitivity = 0.458, specificity = 0.809, area under the curve = 0.629) (Fig. 3).

## Discussion

SSI is one of most serious complications following spine surgery. Previous studies have reported an SSI rate of 0.7–12% in lumbar spine surgeries [24], which is comparable with the 2.04% reported in our study. SSI development is multifactorial, and we found that male sex, age > 60 years, subcutaneous fat thickness, and drainage duration are independent risk factors for SSI development following PLIF, which is in agreement with previous studies [8, 9, 23–25].

LMM, as one of the main components of the trunk extensor muscle, plays a significant role in trunk rotation, spinal stability, lumbar lordosis, and other aspects [26]. Histological studies have verified that LMMs contain numerous type I and type II fibers, suggesting that LMMs serves an important stabilizing function [27]. Previous studies have focused on the association between fat infiltration of LMM and low back pain, and most of concluded that LMM fat infiltration is associated with low back pain [18, 28–31]. The prevalence and severity of LMM fat infiltration increase with age [32, 33]. However, few studies have been performed to examine the association between LMM fat infiltration and SSI development following spine surgery. In our study, for the first time, we found that LMM fat infiltration was significantly increased in the SSI group compared with that in the non-SSI group, indicating that LMM fat infiltration might be a novel significant independent risk factor for SSI development following PLIF.

According to our analysis, LMM fat infiltration and subcutaneous fat thickness may increase the risk for SSI for some reasons. Excess fat tissue in the surgical region may increase the difficulty and duration of the procedure due to lack of adequate exposure of the surgical site [9]. Moreover, fat tissue has less vascularity and lower tissue oxygenation than muscle tissue [34], and the use of retractors to pull apart the paraspinal soft tissue may further decrease blood flow and oxygen tension in fat tissue [24, 35, 36]; this may increase the risk of tissue necrosis and dead space development following wound closure, which have been thought to contribute to the development of postoperative infections [37, 38]. In addition, LMM fat infiltration has its own mechanism through which it increases the risk of SSI. LMM is damaged more than any other muscle in conventional posterior lumbar surgery. Surgeons perform subperiosteal stripping of LMM to expose the operative area; they also cut the multifidus tendinous origin on spinous processes and split the physiological compact binding between muscle fibers and the bony lamina [39]. This may destroy the internal vasculature and tissue structure of LMM. However, the presence of fat infiltration may increase the difficulty for the muscle to attach to cortical bone and may decrease blood flow to fat tissue, which would increase the risk of dead space development and fat tissue ischemic necrosis. Both these would increase SSI risk. In our study, ROC curve analysis also revealed that high LMM fat infiltration was more likely to cause SSI, which supports the above hypothesis.

Furthermore, we showed that increased SSI risk was associated with UTI occurrence. Similarly, another study has demonstrated that UTI is the second most common cause of SSI [40], which is consistent with our findings. A recent study also revealed that operative time (> 3 h) was associated with SSI following spinal surgery [41], and our study further verified that the number of drainage tubes and duration of drainage were associated with the risk of SSI following PLIF. Furthermore, we found that multilevel surgery (≥2 levels) was associated with enhanced risk of SSI following PLIF. Therefore, we speculate that the presence of these risk factors should be minimized during surgery to avoid SSI in patients undergoing PLIF.

In our study, we revealed that although muscle atrophy presents as fat infiltration, there was no correlation between BMI and fat infiltration. Moreover, we revealed that LMM fat infiltration was related to age. Therefore, LMM fat infiltration was a spine-specific SSI risk factor independent of BMI but dependent on age. However, our study has some limitations including its retrospective case–control design. Additionally, a comparison of the postoperative fat infiltration between infected and uninfected patients would have been desirable, although most patients do not undergo MRI following surgery.

## Conclusion

In conclusion, we demonstrated that LMM fat infiltration is a novel risk factor for lumbar postoperative SSI and that body mass distribution better predicted the risk of SSI development than BMI. Finally, increased risk of SSI was associated with male sex, age >60 years, subcutaneous fat thickness, and duration of drainage.

## Supplementary information

**Additional file 1:Figure S1.** Flow chart of grouping.

**Additional file 2:Table S1.** Chi-square test of median percentage of LMM Fat infiltration.

## Data Availability

The datasets used during the current study available from the corresponding author on reasonable request.

## Abbreviations

Alb: Albumin
BMI: Body mass index
HGB: Hemoglobin
LMM: Umbar multifidus muscle
MRI: Magnetic resonance imaging
PLIF: Posterior lumbar interbody fusion surgeries
ROC: Receiver operating characteristic
SSI: Surgery site infection
UTI: Urinary tract infection
