# Supplementary figures and images for: Correlation between lumbar multifidus fat infiltration and lumbar postoperative infection: a retrospective case–control study
Source: BMC Surg. 2020 Feb 24;20:35. doi: 10.1186/s12893-019-0655-9 (PMC7041265; doi:10.1186/s12893-019-0655-9)

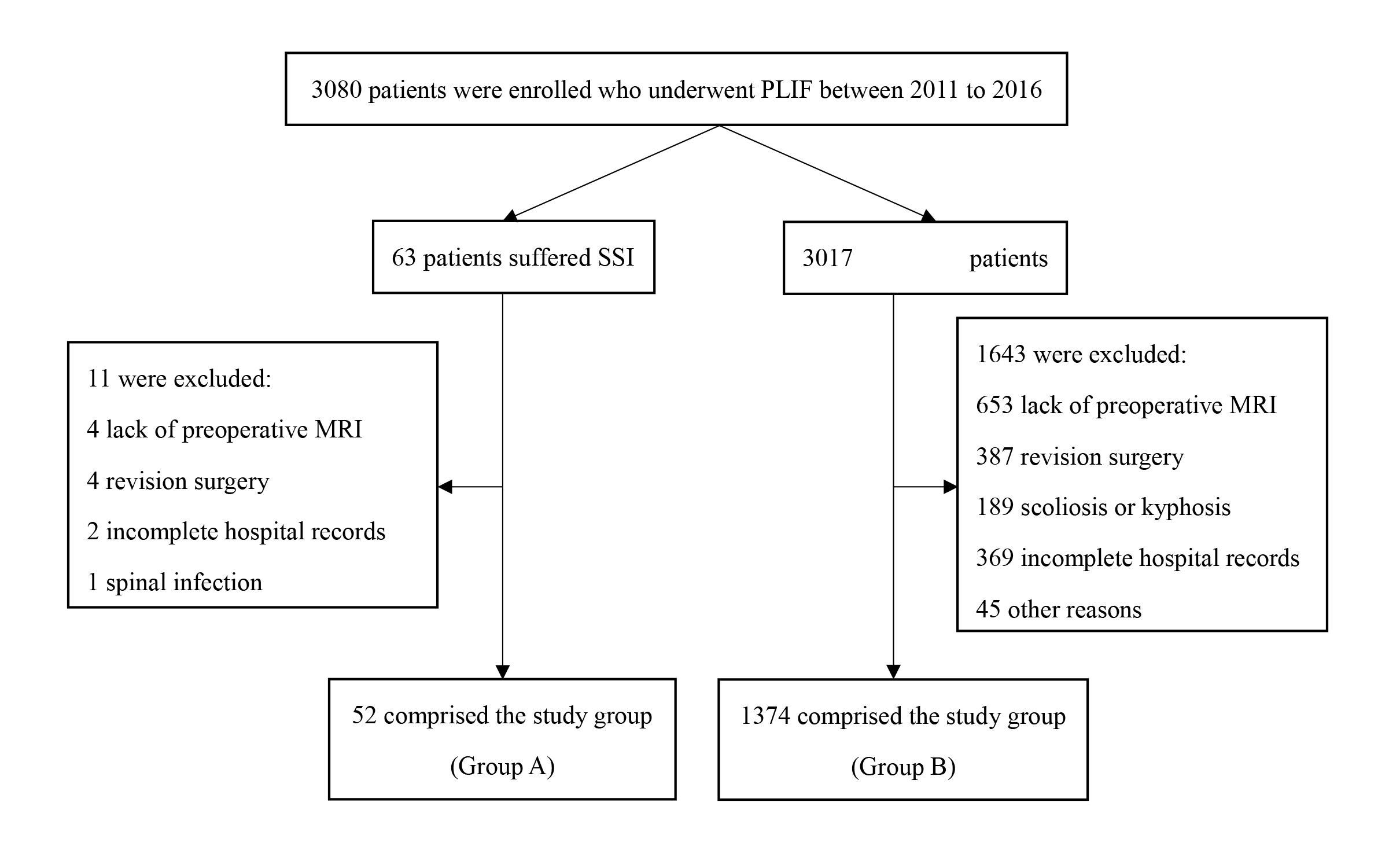

Supplement: Supplementary file 1 — Additional file 1:Figure S1. Flow chart of grouping. [file 12893_2019_655_MOESM1_ESM.tif]
