# Supplementary material for: Correlation between lumbar multifidus fat infiltration and lumbar postoperative infection: a retrospective case–control study
Source: BMC Surg. 2020 Feb 24;20:35. doi: 10.1186/s12893-019-0655-9 (PMC7041265; doi:10.1186/s12893-019-0655-9)
Supplement: Supplementary file 2 — Additional file 2:Table S1. Chi-square test of median percentage of LMM Fat infiltration. [file 12893_2019_655_MOESM2_ESM.docx]

Supplementary table 1. Chi-square test of median percentage of LMM Fat infiltration

|  | **Below the median** | **Above the median** | **Totally** |
| --- | --- | --- | --- |
| **Group A** | 19 (36.54%) | 33 (63.46%) | 52 |
| **Group B** | 787 (57.28%) | 587 (42.72%) | 1374 |
| **Totally** | 806 | 620 | 1426 |

The median percentage of LMM Fat infiltration: 19.60%, χ2=8.770＞χ2_0.05，1_=3.84, *p*=0.003
